# The Angiotensin Converting Enzyme Insertion/Deletion polymorphism is not associated with an increased risk of death or bronchopulmonary dysplasia in ventilated very low birth weight infants

**DOI:** 10.1186/1471-2431-4-26

**Published:** 2004-12-20

**Authors:** Krishna Yanamandra, John Loggins, R John Baier

**Affiliations:** 1 Department of Pediatrics Louisiana State University Health Sciences Center 1501 Kings Highway Shreveport, Louisiana, 71130-3932, USA; 2 Department of Pediatrics and Child Health University of Manitoba WR116 735 Notre Dame Avenue Winnipeg, Manitoba, R3E 0L8, Canada

## Abstract

**Background:**

The ACE gene contains a polymorphism consisting of either the presence (insertion, I) or absence (deletion, D) of a 287 bp alu repeat in intron 16. The D allele is associated with increased ACE activity in both tissue and plasma. The DD genotype is associated with risk of developing ARDS and mortality. The frequency of the D allele is higher in patients with pulmonary fibrosis, sarcoidosis and berylliosis. The role of this polymorphism has not been studied in the development of BPD in the premature newborn.

**Methods:**

ACE I/D genotype was determined in 245 (194 African-American, 47 Caucasian and 4 Hispanic) mechanically ventilated infants weighing less than 1250 grams at birth and compared to outcome (death and/or development of BPD).

**Results:**

The incidence of the D allele in the study population was 0.58. Eighty-eight (35.9%) infants were homozygous DD, 107 (43.7%) were heterozygous ID and 50 (20.4%) were homozygous II. There were no significant differences between genotype groups with respect to ethnic origin, birth weight, gestation, or gender. There was no effect of the ACE I/D polymorphism on mortality or development of BPD (O_2 _on 28 days or 36 weeks PCA). Secondary outcomes (intraventricular hemorrhage and periventricular leukomalacia) similarly were not influenced by the ACE ID polymorphism.

**Conclusions:**

The ACE I/D polymorphism does not significantly influence the development of BPD in ventilated infants less than 1250 grams.

## Background

Prematurely born infants who require mechanical ventilation (MV) frequently develop chronic lung disease known as bronchopulmonary dysplasia (BPD). Oxidant injury, mechanical disruption of the lung, inflammation and subsequent failure of lung development are considered the major mechanisms in the pathogenesis of BPD. The development of BPD is characterized by an initial acute inflammatory component followed by variable degrees of lung fibrosis and failure of alveolar septation, both of which ultimately impair the development of the immature lung [1-5]. The inflammatory component, which consists interstitial and alveolar edema, hyaline membrane formation, epithelial cell necrosis and influx of activated neutrophils, is similar (albeit not identical) to that seen in other forms of acute lung injury (ALl).

There is increasing evidence to support the role for the activation of the renin-angiotensin system (RAS) system during ALI. In ARDS there is an increase in bronchoalveolar lavage and a concomitant decrease in circulating angiotensin converting enzyme (ACE) activity[6,7]. This increase in local ACE activity may influence the course of acute lung injury by its effects on vascular permeability, epithelial cell survival and fibroblast activity [8-12]. Angiotensin-II (AT-II) concentrations are increased in patients with ARDS, consistent with activation of the RAS with ALI[13]. Inhibition of AT-II with type 1 angiotensin receptor antagonists delayed the onset of ARDS and inhibited neutrophil influx in to the lung in experimental models[14]. The role of RAS activation in the lungs of premature infants with respiratory distress syndrome and its evolution into BPD has not been determined.

There is significant variation in circulating ACE activity among individuals, which may be genetically determined. The human ACE gene is located on chromosome 17 (17q23) and contains a polymorphism consisting of either the presence (insertion, I) or absence (deletion, D) of a 287 bp alu repeat in intron 16[15]. The deletion is associated with increased ACE activity in both tissue and in the circulation and accounts for about 47% of the intra-individual variation in plasma ACE activity in Caucasians[15,16]. A role for genetic variation in ACE activity in both acute and chronic lung disease has recently been suggested [17-21]. Higher intrinsic ACE activity (DD genotype) is associated with an increased risk of developing ARDS and other lung diseases [17-21]. The potential role of this polymorphism to influence risk of developing chronic lung disease has not been studied in the premature newborn.

Because of the relationship between inflammation and activation of the RAS and the association with lung fibrosis and adverse outcome in ARDS, the ACE l/D polymorphism may modify risk for the development of chronic lung disease or death in mechanically ventilated VLBW infants. The purpose of this study was to determine if there is a relationship between the ACE I/D polymorphism and respiratory outcomes of death or the development of BPD, in mechanically ventilated very low birth weight (VLBW) infants.

## Methods

Genomic DNA used for this case controlled study was extracted from archival tracheal aspirate (TA) pellets (223 patients) or blood (22 patients) collected prospectively as part of an ongoing study of genetic factors in the development of complications of prematurity. The TAs that were used as a source of genomic DNA were originally collected as part of long term longitudinal studies examining cytokine concentrations and the development of CLD[22,23]. Infants were included in this study if they fulfilled the following inclusion criteria: birth weight less than 1250 grams, mechanical ventilation (MV) during the first week of life, complete clinical data on pulmonary outcome and a genomic DNA sample that could be used for genotyping. Infants were excluded if complete data on pulmonary outcome was not available or suitable DNA was not available. The study was approved by the Institutional Review Board for Human Research at Louisiana State University Health Sciences Center in Shreveport.

Cultures for genital mycoplasmas were performed on samples collected in the first few days of life. Clinical and outcome data were abstracted from the clinical record and included information on respiratory outcome, survival and development of complications of prematurity.

## Laboratory methods

### DNA isolation

Isolation of total DNA from blood or TA pellets was performed using the QIAmp DNA Mini kits™ (Qiagen Incorporated, Chatsworth, CA). Briefly, TA pellets were suspended in 200 μl of sterile phosphate buffered saline by vigorous vortexing, then digested in proteinase K and applied to silica gel spin columns. Columns were washed in the manufacturer's supplied buffers and the total DNA was eluted in 200 μl elution buffer. Blood (200 μl) was extracted similarly to the TA pellets.

### ACE I/D genotyping

ACE I/D polymorphism was performed by microplate PCR method as described previously[24]. (Primers used in the assay: 5'-CTG GAG ACC ACT CCC ATC CTT TCT-3' AND 5'-GAT GTG GCC ATC ACA TTC GTC AGA T-3'. In 10 microliter PCR volume, the following components were added with the final concentration of MgCl_2 _1.5 mM, KCl 50 mM, 5%DMSO, Triton X-100 0.1%, 200 micromolar each of dNTPs, 10 pmol each primer, 1–2 U of Taq polymerase, and 1 microliter of DNA. DNA was denatured at 95°C for 5–10 min, followed by 30 cycles of denaturation at 94° for 60 sec, annealing at 67 for 60 sec, and extension at 72° for 2 minutes, with a final extension at 72° for 7 min. PCR products were separated on 2% agarose gel containing 0.5-microgram/ml ethidium bromide. After gel electrophoresis the bands were visualized by UV-transillumination. The PCR product is a 190 bp fragment in the absence of the insertion (D genotype) and a 490 bp fragment in the presence of the insertion (I genotype).

### Data analysis

Several definitions of BPD have been used through the years. Initially BPD was defined as oxygen dependency at 28 days of age[25,26]. More recently, the use of oxygen dependency at 36 weeks postconceptional age (PCA) has been proposed as a more suitable definition of BPD.[27] Both definitions of BPD predict long term respiratory abnormalities. Data analysis consisted of comparing the frequencies of the ACE I/D genotypes in infants with and without the outcome of interest (supplemental oxygen administration at 28 days or 36 weeks PCA, death or BPD /death before 36 weeks PCA) by Chi Square. All statistical analysis was performed using the SPSS for Windows version 12.0 (SPSS Inc., Chicago, IL). The Student t-test was used to assess normally distributed variables. The Wilcox Rank Sum test was used for analysis of factors that were not normally distributed. A probability value of less than 0.05 was considered statistically significant. The data are presented as mean ± standard error of the mean (SEM).

## Results

Two hundred and forty five (245) patients had complete clinical information on respiratory outcome and genomic DNA available for genotyping. Mean gestational age and birth weight of the study population was 26.4 ± 0.1 weeks and 869 ± 12 grams respectively. One hundred and ninety-four (79%) infants were African-American, 47 (19 %) were Caucasian and 4 (2%) were Hispanic. Male: female ratio was 147:98. All patients required MV at birth and 228 (93%) infants were treated with exogenous surfactant therapy (Survanta^®^, Ross Products Division, Abbott Laboratories, Columbus, OH). Tracheal aspirate cultures obtained during the first few days of life grew *Ureaplasma urealyticum *(Uu) on at least one occasion from 74 of 217 infants cultured (34%) and *Mycoplasma hominis *(Mh) from 29 (13%). One hundred and fifty one (67%) infants were oxygen dependent at 28 days and 55 (25%) were oxygen dependent at 36 weeks PCA. There were 39 (16%) patients who died (from all causes) during their initial hospitalization (24 before 28 days of age and 15 after 28 days).

All 243 infants were genotyped for the ACE I/D polymorphism. The frequency of the D allele in the study population was 0.58. The frequency of the D allele was similar between African-American (0.57) and Caucasian infants (0.59) (p = 0.664). Fifty (20.4%) infants were homozygous II, 107 (43.7 %) were heterozygous ID and 88 (35.9%) were homozygous DD.

Baseline clinical characteristics of birth weight, gestational age, race, gender, TA isolation of Mh, and the need for surfactant replacement were not different between genotype groups (Table 1). Isolation of Ureaplasma urealyticum from the trachea was more frequent in Caucasian infants who had the ACE II genotype (II 55%, ID 9%, DD 22%; p = 0.046). Uu isolation frequencies were similar between genotype groups in African-American infants. Baseline clinical characteristics for Caucasian and African-American infants separately can be found in the online supplement.

**Table 1:** Baseline Clinical Characteristics

	ACE Genotype			
	II (n = 50)	ID (n = 107)	DD (n = 88)	P value
Birth Weight	874 ± 29	876 ± 18	858 ± 20	0.793
Gestation	26.4 ± 0.3	26.5 ± 0.2	26.2 ± 0.2	0.386
Race (Caucasian)	11 (22)	17(16)	19 (22)	0.664
Gender (Males)	28 (56)	59 (55)	59 (67)	0.166
Uu isolated from TA^a^	18/46 (39)	29/88 (33)	27/83 (33)	0.719
Mh isolated from TA^a^	4/46 (9)	14/88 (16)	11/83 (13)	0.507
Surfactant Replacement	48 (96)	99 (93)	81 (92)	0.651

Data are presented as Mean ± Standard error of Mean. Numbers in parenthesis represent percentages.

^a^Not all infants had TA cultures performed for Uu and Mh

Uu *Ureaplasma urealyticum*

Mh *Mycoplasma hominis*

TA Tracheal Aspirate

Clinical characteristics of surviving infants who were or were not oxygen dependent at days are shown in Table 2. Infants who were oxygen dependent at 28 days were less mature, of lower birthweight, more likely to have TA isolation of Uu or Mh, and more likely to have received surfactant replacement therapy than those weaned from oxygen by 28 days of age. Ethnic groups and gender were not different between outcome groups. Infants who died or who were oxygen dependent at 36 weeks PCA (BPD) were similarly less mature, of lower birthweight, and were more likely to have received surfactant replacement therapy than surviving infants without BPD (Table 3). Isolation of either Uu or Mh from the TA had no influence on this outcome.

**Table 2:** Comparison of Infants Oxygen Dependent at 28 days

	No Oxygen at 28 days (n = 75)	Oxygen at 28 days (n = 151)	P value
Gestation (weeks)	27.4 ± 0.1	26.0 ± 0.1	<0.001
Birth Weight (grams)	996 ± 18	826 ± 14	<0.001
Race (Caucasian)	20 (27)	26 (17)	0.103
Gender (Males)	39 (52)	95 (63)	0.102
Surfactant therapy	75 (87)	144 (95)	0.020
Ureaplasma isolated^a^	16/67 (24)	55/134 (41)	0.016
Mycoplasma isolated^a^	4/67 (6)	23/134 (17)	0.028
IVH^b^	8/74 (11)	55/151 (36)	<0.001
IVH = Grade 3^b^	4/74 (5)	35/151 (23)	<0.001
PVL^b^	2/74 (3)	15/151 (10)	0.054

Data are presented as Mean ± Standard error of Mean. Numbers in parenthesis represent percentages.

^a^Not all infants had TA cultures performed for Uu and Mh

^b^Not all infants had cranial US evaluations

Uu *Ureaplasma urealyticum *Mh *Mycoplasma hominis *IVH Intraventricular Hemorrhage PVL Periventricular leukomalacia TA Tracheal Aspirate

**Table 3:** Clinical Characteristics of Infants who Died or Developed BPD

	Survival without BPD (n = 162)	Death or BPD (n = 83)	P value
Gestation (weeks)	26.6 ± 0.1	25.8 ± 0.2	<0.001
Birth Weight (grams)	925 ± 14	760 ± 17	<0.001
Race (Caucasian)	33 (20)	14 (17)	0.737
Gender (Males)	96 (60)	50 (60)	0.926
Surfactant therapy	147 (91)	81 (98)	0.046
Ureaplasma isolated^a^	49/144 (34)	25/73 (34)	0.974
Mycoplasma isolated^a^	20/144 (14)	9/64 (12)	0.750
IVH^b^	31/161 (19)	41/80 (52)	<0.001
IVH = Grade 3^b^	15/161 (9)	31/80 (39)	<0.001
PVL^b^	8/161 (5)	10/80 (13)	0.033

Data are presented as Mean ± Standard error of Mean. Numbers in parenthesis represent percentages.

^a^Not all infants had TA cultures performed for Uu and Mh

^b^Not all infants had cranial US evaluations

Uu *Ureaplasma urealyticum *Mh *Mycoplasma hominis *IVH Intraventricular Hemorrhage PVL Periventricular leukomalacia TA Tracheal Aspirate

Because the ACE I/D polymorphism may have different functional effects on plasma and tissue ACE activities in different ethnic groups, we analyzed the effects of ACE on the incidence of BPD separately for Caucasian and African-American infants. (The effects of the ACE I/D polymorphism on the incidence of BPD and other outcomes for the combined group can be found in the online data supplement). Table 4 shows the effects of ACE genotype on outcomes in ventilated Caucasian infants less than 1250 grams. There was no significant effect of ACE genotype on mortality, oxygen dependency at either 28 days or 36 weeks PCA or the combined outcome of death or BPD. The incidence of periventricular leukomalacia (PVL) was significantly higher in Caucasian infants with the II genotype. The incidence and severity of intraventricular hemorrhage (IVH) was not affected by ACE genotype.

**Table 4:** Effect of ACE genotype on Outcomes in Caucasian Infants

	ACE Genotype			
	II (n = 11)	ID (n = 17)	DD (n = 19)	P value
Oxygen at 28 days	6/11 (55)	10/15 (67)	9/18 (47)	0.620
Oxygen at 36 weeks PCA	3/10 (30)	4/15 (27)	3/18 (17)	0.674
Death <28 days	0 (0)	1 (16)	1 (5)	0.724
Death or Oxygen at 36 weeks	4 (36)	6 (35)	4 (21)	0.558
Death ≥ 28 days	3 (27)	1 (6)	0 (0)	0.039
IVH^a^	2/10 (20)	6/17 (35)	1/19 (5)	0.076
IVH ≥ Grade 3^a^	2/10 (20)	3/17 (18)	1/19 (5)	0.415
PVL^a^	3/10 (30)	0/17 (0)	1/19 (5)	0.022

Numbers in parenthesis represent percentages.

^a^Not all infants had cranial US evaluations

PCA Postconceptional age IVH Intraventricular Hemorrhage PVL Periventricular leukomalacia

The effects of ACE genotype on outcome in ventilated African-American infants less than 1250 grams are shown in Table 5. There was no significant effect of ACE genotype on mortality, oxygen dependency at either 28 days or 36 weeks PCA or the combined outcome of death or BPD. The incidence of PVL was not affected by ACE genotype in African-American infants. Similar to that observed in Caucasian infants, there was no apparent effect of the ACE I/D polymorphism on the incidence and severity of IVH in African American infants.

**Table 5:** Effect of ACE genotype on Outcomes in African-American Infants

	ACE Genotype			
	II (n = 39)	ID (n = 88)	DD (n = 66)	P value
Oxygen at 28 days	24/36 (67)	57/82 (70)	43/59 (73)	0.805
Oxygen at 36 weeks PCA	7/34 (21)	22/79 (28)	16/58 (28)	0.698
Death <28 days	3 (8)	6 (7)	8 (12)	0.517
Death or Oxygen at 36 weeks	12 (31)	31 (35)	25 (37)	0.792
Death ≥ 28 days	3 (8)	7 (9)	4 (7)	0.925
IVH^a^	14/38 (37)	23/86 (27)	25/67 (37)	0.311
IVH ≥ Grade 3^a^	7/38 (18)	15/86 (17)	17/67 (25)	0.438
PVL^a^	1/38 (3)	5/86 (6)	8 (12)	0.145

Numbers in parenthesis represent percentages.

^a^Not all infants had cranial US evaluations

PCA Postconceptional age IVH Intraventricular Hemorrhage PVL Periventricular leukomalacia

Since birth weight and gestation are the primary determinants of adverse outcome, analysis was repeated in both infants greater than 750 grams and infants ≤ 750 grams. There was no significant effect of ACE genotype when this subgroup analysis was performed (data not shown).

## Discussion

The RAS is activated during lung injury and plays a role in several pathological processes. In addition to the pulmonary endothelium, respiratory epithelium also possesses significant ACE activity. Fas-induced alveolar epithelial cell apoptosis is dependent on local AT-II production and interaction with it receptor.[10,11] Further, AT-II is mitogenic for lung fibroblasts and aberrant AT-II production has been linked with some forms of pulmonary fibrosis[12,18,28-30]. Inhibition of AT-II with type 1 angiotensin receptor antagonists delayed the onset of ARDS and inhibited neutrophil influx into the lung in experimental models[14]. In adults, there is an increase in bronchoalveolar lavage ACE activity and AT-II during ALI, however the contribution of activation of the RAS to neonatal lung injury has received little study[6,13].

The frequency of the D allele in our study population was not different than reported in our local population or for other groups[16,24,31]. The ACE D allele is common with a frequency approximately 50–60% in Caucasians and 60–65% in African-Americans. This suggests that the D allele is neither a risk factor nor a protective factor for either premature birth or the need for mechanical ventilation. We did not study early cardiovascular adaptation or record measures of early illness severity, as did Harding et al[32] In that study the DD genotype was associated with a worse cardiovascular adaptation. In our study, the infants are much more immature, of lower gestational age and almost universally had hyaline membrane disease.

In our study population, the ACE ID polymorphism was not associated with an altered risk for the development of BPD, oxygen dependency at 28 days, death (early, late or total mortality) or composite outcomes (BPD or death) suggesting that factors other than genetic variation at this locus contribute to these outcomes. This is in contrast to that seen in adults with ARDS or other lung disorders such as pulmonary fibrosis, sarcoidosis and berylliosis where the D allele is associated with disease [18-21].

Indeed genetic factors may not have a large influence in a disease (BPD) where maturity at birth has such an overwhelming influence. Our previous study in this population failed to find any association of the tumor necrosis factor-α-308 G/A, MCP-1-2518 A/G or transforming growth factor-β_1 _+915 G/C SNPs on the development of BPD[33] Additionally we have found that the IL-10-1082 G/A SNP has no effect on the incidence of BPD (Yanamandra et al, Pediatric Pulmonology, in press).

The finding of increased incidence of PVL in Caucasian infants with the II genotype may be due more to the greatly increased incidence of *Ureaplasma urealyticum *colonization in these infants. Chorioamnionitis, which is highly correlated with isolation of Uu, is a risk factor for PVL.[34] Because of the retrospective nature of this study we were not able to systematically review placental pathology.

Our study has several limitations. The ACE I/D polymorphism has only been demonstrated to be functional in Caucasians.[16] Data linking this polymorphism and ACE activity in other ethnic groups are either lacking or suggests the polymorphism is non functional (African-Americans)[31]. If this were true, then one would expect to find no effect of the ACE I/D polymorphism on outcomes in African-American infants. The numbers of Caucasian infants in this study are few and an effect of the ACE I/D polymorphism on the incidence of chronic lung disease or other outcomes may not be detected. Thus it may be relevant to examine the contribution of the ACE I/D polymorphism to chronic lung disease in a larger cohort of Caucasian infants.

The observations of this retrospective case controlled study are also limited somewhat by selection bias. Because only infants who were mechanically ventilated were included, the true impact of this polymorphism on the complications of prematurity may be underestimated. Since this was a retrospective study using stored material no attempt was made to correlate phenotype with genotype (ie TA ACE measurements).

Lung injury and the subsequent maladaptive repair process that leads to the development of BPD is complex with a great many factors that interplay to determine outcome. It is very likely that other genetic play a role in determining the risk of poor outcome. Polymorphisms in cytokines, their receptors, bacterial pattern recognition molecules, surfactant proteins, and heme oxygenase-1 are known to alter the course of other pulmonary diseases and should be examined as to their potential role in the development of BPD in the premature infant.

## Conclusions

The ACE I/D polymorphism does not significantly influence the development of BPD in ventilated infants less than 1250 grams.

## Competing interests

The author(s) declare that they have no competing interests.

## Authors' contributions

RJB conceived and organized the study, prepared the manuscript and performed the statistical analyses. KY performed the genotyping, and assisted with manuscript preparation. JL oversaw collection tracheal aspirates, assisted with recruitment of subjects into the original studies, and assisted with editing of the manuscript. All authors read and approved the final manuscript.

## Pre-publication history

The pre-publication history for this paper can be accessed here:



## Supplementary Material

Additional File 1 Contains Tables showing baseline clinical characteristics of African American and Caucasian infants separately by Genotype group Click here for file
