# Supplementary material for: The Angiotensin Converting Enzyme Insertion/Deletion polymorphism is not associated with an increased risk of death or bronchopulmonary dysplasia in ventilated very low birth weight infants
Source: BMC Pediatr. 2004 Dec 20;4:26. doi: 10.1186/1471-2431-4-26 (PMC544573; doi:10.1186/1471-2431-4-26)
Supplement: Additional File 1 — Contains Tables showing baseline clinical characteristics of African American and Caucasian infants separately by Genotype group [file 1471-2431-4-26-S1.doc]

Supplemental Tables for The Angiotensin Converting Enzyme Insertion/Deletion Polymorphism is not associated with an increased risk of death or Bronchopulmonary Dysplasia in ventilated Very Low Birth Weight infants.

**Table 1S**

Baseline Clinical Characteristics
(Caucasians)

|  | ACE Genotype | | | |
| --- | --- | --- | --- | --- |
|  | II  (n=11) | ID  (n=17) | DD  (n=19) | P value |
| Birth Weight | 913±58 | 950±52 | 922±44 | 0.865 |
| Gestation | 26.5±0.5 | 27.1±0.4 | 26.6±0.4 | 0.577 |
| Gender (Males) | 6 (55) | 7 (55) | 13 (68) | 0.260 |
| Uu isolated from TA^a^ | 6/11 (55) | 1/11 (9) | 4/18 (22) | 0.046 |
| Mh isolated from TA^a^ | 0/11 (0) | 1/11 (9) | 1/18 (6) | 0.613 |
| Surfactant Replacement | 9 (82) | 17 (100) | 19 (100) | 0.033 |

^a^Not all infants had TA cultures performed for Uu and Mh

Uu *Ureaplasma urealyticum* Mh *Mycoplasma hominis*

TA Tracheal Aspirate

**Table 2S**

Baseline Clinical Characteristics
(African-American)

|  | ACE Genotype | | | |
| --- | --- | --- | --- | --- |
|  | II  (n=39) | ID  (n=88) | DD  (n=67) | P value |
| Birth Weight | 864±35 | 865±19 | 838±22 | 0.665 |
| Gestation | 26.3±0.3 | 26.4±0.2 | 26.0±0.2 | 0.394 |
| Gender (Males) | 22 (56) | 50 (57) | 44 (66) | 0.479 |
| Uu isolated from TA^a^ | 12/35 (34) | 27/76 (36) | 22/63 (35) | 0.992 |
| Mh isolated from TA^a^ | 4/35 (11) | 13/76 (17) | 10/63 (16) | 0.741 |
| Surfactant Replacement | 39 (100) | 80 (90) | 67 (100) | 0.123 |

^a^Not all infants had TA cultures performed for Uu and Mh

Uu *Ureaplasma urealyticum* Mh *Mycoplasma hominis*

TA Tracheal Aspirate

**Table 3S**

**Effect of ACE genotype on Outcomes**

**(All Infants)**

|  | ACE Genotype | | | |
| --- | --- | --- | --- | --- |
|  | II  (n=50) | ID  (n=107) | DD  (n=88) | P value |
| Oxygen at 28 days | 30/47 (64) | 69/99 (70) | 52/80 (65) | 0.712 |
| Oxygen at 36 weeks PCA | 10/44 (23) | 26/95 (27) | 19/79 (24) | 0.805 |
| Death <28 days | 4 (8) | 10 (9) | 10 (11) | 0.798 |
| Death or Oxygen at 36 weeks | 16 (32) | 38 (36) | 29 (33) | 0.887 |
| Death ≥ 28 days | 5/48 (10) | 6/104 (6) | 4/84 (5) | 0.417 |
| IVH^a^ | 16/48 (33) | 30/104 (29) | 26/88 (30) | 0.849 |
| IVH ≥ Grade 3^a^ | 9/48 (19) | 19/104 (18) | 18/88 (21) | 0.915 |
| PVL^a^ | 4/48 (8) | 5/104 (5) | 9 (11) | 0.321 |

Numbers in parenthesis represent percentages.

^a^Not all infants had cranial US evaluations

PCA Postconceptional age IVH Intraventricular Hemorrhage

PVL Periventricular leukomalacia
